# Peripheral Nerve Stimulation in Chronic Knee Pain: A Case Series

**DOI:** 10.7759/cureus.50127

**Published:** 2023-12-07

**Authors:** Timothy D Kelly, Michael L Pazzol, Raheleh Rahimi Darabad

**Affiliations:** 1 Department of Emergency Medicine Residency, Indiana University School of Medicine, Indianapolis, USA; 2 Department of Anesthesiology and Perioperative Medicine, University of Rochester School of Medicine and Dentistry, Rochester, USA; 3 Department of Anesthesiology, Indiana University School of Medicine, Indianapolis, USA

**Keywords:** neuromodulation, interventional pain management, ultrasound, chronic knee pain, peripheral nerve stimulation

## Abstract

Introduction

Chronic knee pain is increasing in prevalence and is associated with substantial limitations in functional mobility. Peripheral nerve stimulation (PNS) has been increasingly used to treat various chronic pain conditions. However, there is a paucity of research exploring the potential therapeutic benefit of PNS for chronic knee pain.

Methods

This research is a retrospective case series of all patients who received PNS for the treatment of chronic knee pain performed at a single-center academic medical institution between March 2021 and June 2022. The primary outcome was percent pain reduction six months after implantation. Outcome data was obtained via chart review and phone calls to patients. Secondary outcomes included percent pain reduction two weeks and two months after implantation and adverse medical events related to the procedure and nerve stimulation.

Results

Fourteen individual patients received PNS for chronic knee pain during the study period. Three of these patients received bilateral PNS for a total of 17 cases. The mean percent pain reduction six months after implantation was 52% (SD=28.2) (N=12). A total of 75.0% of participants (9/12) reported *≥*50% reductions in pain six months after implantation. No adverse events were reported relating to the implantation procedure and/or nerve stimulation.

Conclusion

PNS is a safe and efficacious treatment modality for chronic knee pain with demonstrated long-term benefit. Further research should clarify patient factors associated with improved treatment response.

## Introduction

Chronic knee pain can be a debilitating medical condition that negatively impacts patients’ quality of life and functional mobility [1]. Even in its early stages, chronic knee pain is associated with meaningful reductions in patient activity and behavioral modification [2]. Furthermore, chronic knee pain is additionally associated with substantial economic burdens borne both by the healthcare system and the patient [3, 4]. The high clinical morbidity associated with chronic knee pain requires novel and creative therapeutic approaches to better address this frequently disabling condition. Peripheral nerve stimulation (PNS) is a relatively new technique for treating chronic pain and has been shown to be efficacious in treating chronic pain of various etiologies and locations [5, 6]. Given its favorable side-effect profile and expanding indications for use [7], PNS has been increasingly utilized by interventional pain physicians to treat refractory pain conditions [8-10]. Recently, PNS has been applied to treating chronic knee pain and has shown promising, if limited, results [11, 12]. Much of the literature surrounding PNS and chronic knee pain, however, has focused on the role of PNS in peri-operative/post-operative pain following knee surgery [13]. However, the potential utility of PNS in treating chronic knee pain in other clinical settings has received less focus. In this original investigation, we present a retrospective case series of patients with chronic knee pain who failed conservative measures and received PNS in clinical settings apart from the post-operative period. We aim to provide important data regarding the potential utility of PNS in the management of chronic knee pain.

## Materials and methods

This investigation is a retrospective case series of patients who received PNS to treat chronic knee pain. All patients received care from a board-certified Pain Medicine physician (Darabad RR) at a single-center, tertiary academic medical center. PNS implantations were performed with ultrasound guidance. This study received Institutional Review Board (IRB) approval from the Indiana University IRB (approval number: 13029). All patients received the SPRINT® PNS System (SPR Therapeutics, Cleveland, OH, USA), a minimally invasive implant designed for short-term use. The SPRINT® PNS system consists of one or two leads inserted percutaneously, an external pulse generator delivering continuous therapy with a rechargeable battery pack, a mounting pad holding the pulse generator, and a handheld remote for stimulation level control. Due to the temporary nature of the treatment, patients can maintain most of their daily activities with minimal restrictions. To insert the SPRINT® PNS system, a manufacturer-designed percutaneous sleeve is inserted under ultrasound (as demonstrated in this case series) or fluoroscopic guidance. After achieving the appropriate degree of nerve stimulation, a Microlead is inserted and tested again. The conduit is then removed, leaving the Microlead behind. The lead(s) remain in place for two months before removal. A two-lead system provides more robust stimulation compared to a one-lead system. For chronic knee pain, potential nerve targets include femoral and saphenous nerves, depending on the pain distribution. If complete coverage is achieved with one nerve, two leads may be inserted on the same nerve for increased stimulation robustness and as an alternative lead in case of lead migration. In cases of pain in the anterior and medial aspects of the knee, a first lead may target the femoral nerve, and a second lead may target the saphenous nerve. Genicular nerves are also potential targets, with fluoroscopic guidance more commonly used. Given the significant pain relief observed when targeting femoral and saphenous nerves, authors have more commonly utilized this technique compared to targeting genicular nerves. Data was collected through the electronic medical record (EMR) and direct phone calls to patients. For each study participant, investigators used the EMR to gather demographic information, knee pain characteristics, pain scores at prescribed time intervals, and information regarding adverse medical events and additional performed procedures/surgeries. Given that the EMR was frequently incomplete, investigators called each patient up to three times during normal business hours to gather missing data when able. In rare instances where information provided via telephone differed from that noted in the EMR, the patient’s verbal response was preferentially used. The primary outcome of the case series was a perceived percentage reduction in chronic pain six months after PNS implantation. Clinically significant reductions in pain were a priori determined to be percentage reductions of 50% or greater, based on published benchmarks [14-16]. Due to the limitations of our study’s retrospective design, there were instances where the perceived percentage reduction in pain was not recorded in the EMR, and the patient could not be reached by phone for clarification. In these cases, perceived percentage reductions in pain were extrapolated based on the 11-point numerical pain rating scores (NRS) listed in the EMR. The NRS is a quantitative measure of pain frequently used in chronic pain research and has been validated in multiple studies across diverse populations [17-19]. Secondary outcomes included adverse medical events and the need for other surgeries/procedures relating to chronic knee pain. Adverse medical events were defined as serious bacterial infections (such as cellulitis, sepsis, etc.) relating to PNS implantation, vascular injury, or functional neurological impairment. Every patient who received PNS for chronic knee pain between March 2021 and June 2022 was included in the overall investigation. However, primary and secondary outcomes are reported for participants whose data was available. In rare cases in which a patient underwent bilateral PNS implantation for chronic knee pain, each implantation was treated as a separate study encounter.

## Results

During the 15-month study period, 14 individual patients received PNS implantation to treat chronic knee pain. Three of these individuals received bilateral PNS implants for chronic knee pain; thus, there were 17 separate study encounters. Five patients (5/17, 29.4%) were male, and twelve (12/17, 70.6%) were female. Table 1 lists demographic information, chronic knee pain location, specific nerves targeted, and number of implants for the 17 study participants.

**Table 1:** Participants' demographic information.

Case Number	Age	Sex	Location of pain	Nerve(s) targeted
1	60	Female	Anterior	Femoral
2	60	Female	Anterior	Femoral
3	68	Male	Medial	Femoral, Saphenous
4	64	Female	Anterior	Saphenous x 2
5	48	Female	Anterior	Femoral, Saphenous
6	65	Male	Anterior	Femoral
7	65	Male	Anterior	Femoral
8	47	Female	Medial	Femoral, Saphenous
9	45	Female	Anterior	Femoral x 2
10	45	Female	Anterior	Femoral x 2
11	65	Female	Anterior	Femoral x 2
12	69	Female	Diffuse	Femoral x 2
13	34	Male	Anterior	Femoral x 2
14	53	Female	Medial/Inferior	Femoral x 2
15	57	Female	Anterior	Femoral
16	73	Female	Anterior	Femoral
17	25	Male	Anterior/Posterior	Sciatic x 2

The etiology of chronic knee pain of the included participants is shown in Table 2. Four participants (4/17, 23.5%) previously underwent surgical intervention of their affected knee, either via total knee arthroplasty (TKA) (N=3) and/or orthopedic reconstructions following trauma (N=1). 

**Table 2:** Etiology of chronic knee pain.

Etiology of chronic knee pain	Number of participants
Osteoarthritis	11/17 (64.7%)
Chronic knee pain after TKA	3/17 (17.6%)
Chronic knee pain after trauma	2/17 (11.8%)
Sciatic neuralgia	1/17 (5.9%)

**Figure 1:**
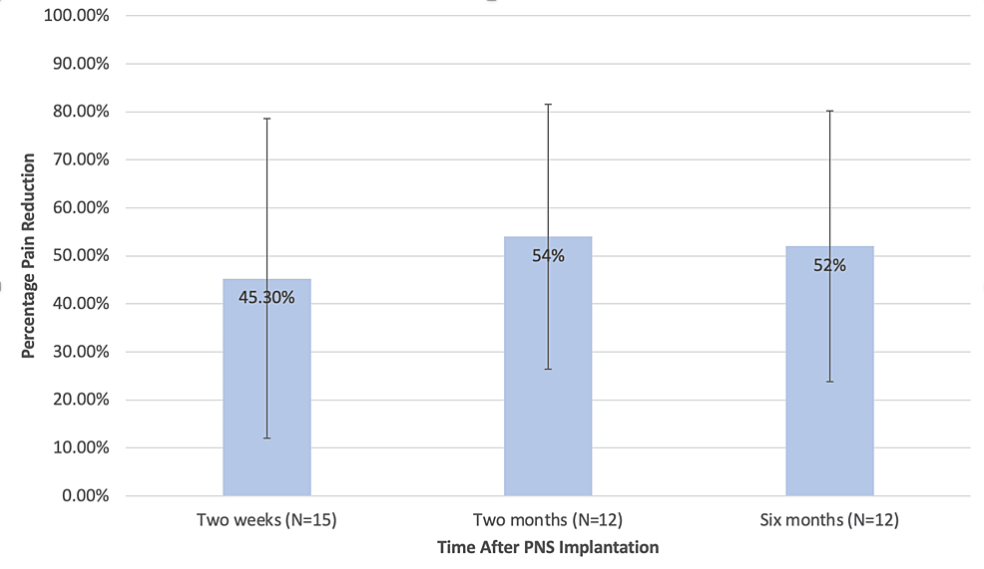
Mean percentage reduction in pain following PNS implantation. PNS: Peripheral nerve stimulation.

The clinical histories of many of the participants shared some common themes. One of the participants was a 60-year-old female with a past medical history of obesity, chronic obstructive pulmonary disease (COPD), and obstructive sleep apnea (OSA) whose symptoms of bilateral knee osteoarthritis severely impacted her functional mobility and quality of life. Prior to undergoing PNS, she had tried multiple conservative therapies without relief, including physical therapy, nonsteroidal anti-inflammatory drugs (NSAIDs), opioid medications, nerve-blocking agents, and corticosteroid injections. She was a poor surgical candidate, given her medical comorbidities, and was instead referred for PNS. Likewise, another participant was a 69-year-old female with a past medical history of lymphoma, asthma, and hypertension who also struggled with intractable right knee pain due to osteoarthritis. Her symptoms persisted despite multiple rounds of physical therapy, NSAIDS, opioid medications, and corticosteroid injections. The patient preferred to forgo surgical intervention of the affected knee, at which point she was referred for PNS. The mean perceived percentage reduction in chronic pain was 45.3% (SD=33.3) (N=15) at two weeks, 54% (SD=27.6) (N=12) at two months, and 52% (SD=28.2) (N=12) at six months after PNS implantation. Figure 1 shows these perceived percentage pain reductions at the two-week, two-month, and six-month intervals. Number of patients for whom data is available decreased during the study period as patients were lost to follow-up. 

**Figure 2:**
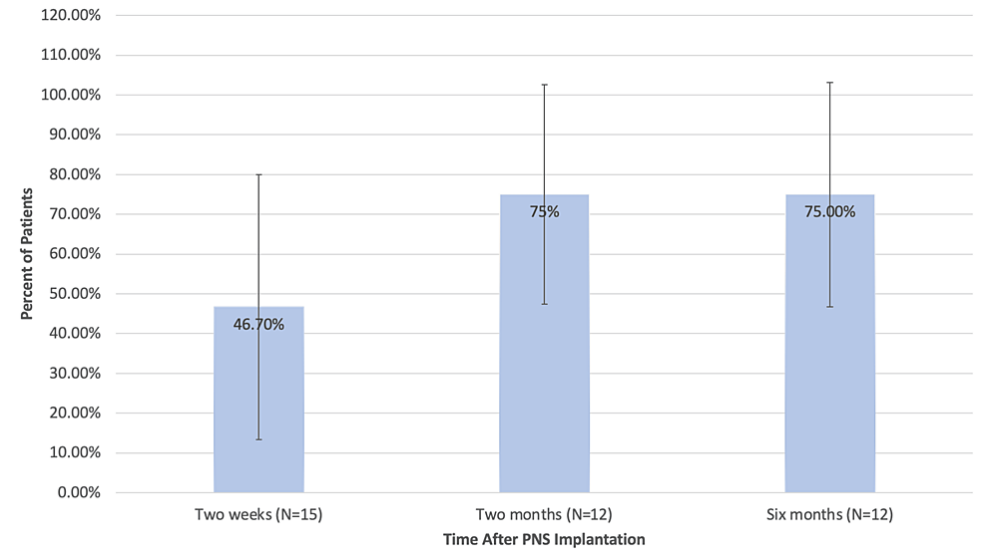
Percentage of patients with clinically significant pain reductions.

Six months after PNS implantation, 75.0% of participants (9/12) reported clinically significant reductions in pain (defined a priori as ≥50% reduction in pain). Interestingly, increasing proportions of participants endorsed clinically significant reductions in pain as time went on, as depicted in Figure 2. Table 3 shows the specific percentage reduction in pain six months after PNS implantation amongst treatment responders, i.e., participants with ≥ 50% reduction in pain.

**Table 3:** Percentage reduction in pain amongst treatment responders at six months.

Case Number	Percent Pain Reduction
1	60%
2	60%
3	90%
6	65%
7	65%
11	70%
12	75%
14	60%
16	50%

None of the participants experienced an adverse medical event relating to their PNS implantation during the study period, although one participant died due to COVID-19 pneumonia. Only one participant (1/17, 5.9%) underwent a surgical operation relating to their chronic knee pain (total knee arthroplasty) during the six-month follow-up period. 

## Discussion

This investigation provides meaningful contributions to the growing body of evidence that PNS is a safe and efficacious treatment modality for managing chronic knee pain [11-13]. Notably, our investigation is one of the first to suggest that PNS for chronic knee pain may be effective in clinical settings apart from the immediate peri-operative period. Importantly, this investigation is one of the most extensive known case series to date that explores patient outcomes after PNS implantation for chronic knee pain [11-13, 20, 21]. Although limited to retrospective observational data collection, our study demonstrates that a majority of patients (75.0%) in whom data was available experienced substantial reductions in clinical pain scores (defined as ≥50% pain relief) six months after undergoing PNS implantation. Interestingly, the average percentage of pain relief two weeks after PNS implantation was similar to that of pain relief six months after PNS implantation. Overall, these results suggest that PNS implantation has the potential for sustained reductions in chronic knee pain that persists months after implantation. Given that our case series lacks a control group, making inferences regarding a possible placebo effect is challenging. That being said, a previous comparison of PNS vs. placebo for the treatment of chronic neuropathic postamputation pain found that only 14% of patients receiving placebo experienced substantial reductions in clinical pain scores (similarly defined as ≥50% pain relief) [22]. This estimated placebo effect is well below the observed treatment effect in our population. The presented investigation is notable in other ways, as well. Given the relative size of this case series, the lack of any serious adverse events lends further evidence that PNS is a safe treatment modality. Furthermore, our study included every patient who received PNS for chronic knee pain at our institution regardless of age, sex, chronic pain etiology, and/or specific location (i.e., anterior knee, medial knee, etc.). These results suggest that PNS implantation may be useful in treating chronic knee pain for diverse patients. One of the most significant aspects of the presented investigation is that relatively few patients who received PNS for chronic knee pain later underwent surgical intervention during the 15-month study period, albeit a small number of participants had already undergone surgical intervention before PNS implantation. This is especially important for patients with chronic knee pain in whom surgical intervention is contraindicated due to high-risk surgical comorbidities, especially given that functional limitations from chronic knee pain are more common in patients with medical comorbidities [23]. Even though our investigation does not have the methodological ability to demonstrate that PNS for chronic knee pain reduces the need for surgical intervention, the relatively small number of patients who went on to receive surgical intervention is notable.

Our findings provide directions for additional scholarship. Future investigations would do well to clarify what subset of patients experience the greatest functional benefit from PNS implantation as well as explore the relationship between PNS efficacy and type of chronic pain syndrome (i.e., neuropathic, musculoskeletal, etc.). In the present study, 25% of patients did not achieve clinically significant reductions in pain. While our study design is unable to provide why this is so, we hypothesize that PNS efficacy in treating chronic knee pain may be impacted by pre-procedural pain severity. This question is ripe for future scholarship. Other promising avenues of future study concern the ability of PNS implantation to delay and/or defer surgical intervention, the ability of PNS to decrease the need for prescribed analgesics, and the most effective duration of nerve stimulation. Ideally, these investigations would include blinding, comparative interventions, and a randomized control trial. This study has important limitations. The most important of these limitations is the retrospective nature of the investigation. Like all retrospective observational studies, the presented study is limited in its external validity by its lack of blinding and comparative interventions. Similarly, this study is subject to patient recall bias and is hindered by an imperfect ability to collect complete patient data. Frequently, missing data was due to patients being lost to follow-up. Reasons why patients were lost to follow-up were not immediately clear, except for one deceased patient. Finally, our study is not designed nor powered to prove that PNS for chronic knee pain reduces and/or prevents later surgical intervention. Nonetheless, we believe that this study provides important data surrounding the effectiveness of PNS for chronic knee pain and will encourage future robust investigations.

## Conclusions

PNS demonstrates substantial promise for the treatment of chronic knee pain. Most patients who received PNS for chronic knee pain reported clinically significant reductions in chronic pain six months after PNS implantation. PNS may be a beneficial treatment modality for those who are poor operative candidates and/or decline surgical intervention. Future prospective investigations are needed to clarify better the optimal role of PNS in the treatment of chronic knee pain.
